# Proteomic and metabolomic analyses provide insight into production of volatile and non-volatile flavor components in mandarin hybrid fruit

**DOI:** 10.1186/s12870-015-0466-9

**Published:** 2015-03-06

**Authors:** Qibin Yu, Anne Plotto, Elizabeth A Baldwin, Jinhe Bai, Ming Huang, Yuan Yu, Harvinder S Dhaliwal, Frederick G Gmitter

**Affiliations:** University of Florida, Institute of Food and Agricultural Sciences, Citrus Research and Education Center, Lake Alfred, FL 33850 USA; USDA-ARS Horticultural Research Laboratory, Fort Pierce, FL 34945 USA; College of Agriculture, Punjab Agricultural University, Ludhiana, Punjab 141004 India

**Keywords:** Apocarotenoid volatiles, Carotenoids, Sesquiterpene synthase, Citrus, Gene expression

## Abstract

**Background:**

Although many of the volatile constituents of flavor and aroma in citrus have been identified, the knowledge of molecular mechanisms and regulation of volatile production are very limited. Our aim was to understand mechanisms of flavor volatile production and regulation in mandarin fruit.

**Result:**

Fruits of two mandarin hybrids, Temple and Murcott with contrasting volatile and non- volatile profiles, were collected at three developmental stages. A combination of methods, including the isobaric tags for relative and absolute quantification (iTRAQ), quantitative real-time polymerase chain reaction, gas chromatography, and high-performance liquid chromatography, was used to identify proteins, measure gene expression levels, volatiles, sugars, organic acids and carotenoids. Two thirds of differentially expressed proteins were identified in the pathways of glycolysis, citric acid cycle, amino acid, sugar and starch metabolism. An enzyme encoding valencene synthase gene (*Cstps1*) was more abundant in Temple than in Murcott. Valencene accounted for 9.4% of total volatile content in Temple, whereas no valencene was detected in Murcott fruit. Murcott expression of *Cstps1* is severely reduced.

**Conclusion:**

We showed that the diversion of valencene and other sesquiterpenes into the terpenoid pathway together with high production of apocarotenoid volatiles might have resulted in the lower concentration of carotenoids in Temple fruit.

**Electronic supplementary material:**

The online version of this article (doi:10.1186/s12870-015-0466-9) contains supplementary material, which is available to authorized users.

## Background

Fruit volatiles are essential components of fruit flavor, have defense mechanisms against biotic and abiotic stresses, and contribute to various physiological and ecological functions during plant development [1]. Flavor in mandarin fruit is the result of a combination of sugars (glucose, sucrose and fructose), acids (citric and malic), flavonoids, limonoids, and volatile compounds [2]. Volatiles in mandarin fruit belong to several chemical families such as terpenes, hydrocarbons, aldehydes, esters, alcohols, ketones and sulfur compounds [3]. Terpenoids play a central role in generating the chemical diversity, and accounted for 85–95% of volatiles in tangerine fruit [4]. Most volatiles are derived from a diverse set of non-volatile precursors, simple or complex molecules including amino acids, fatty acids, carbohydrates and carotenoids, which can be grouped into four biosynthetic classes: terpenoids, fatty acids, branched-chain amino acids and aromatic amino acids such as phenylalanine [5]. Virtually all of these precursors are essential human nutrients [6].

Breeding for improvement of fruit flavor is a very challenging task when using classical breeding methods due to the difficulty of scoring and quantifying such a complex trait. The presence of a single volatile molecule, even at a relatively high level, does not mean that it contributes to either flavor or liking [7]. To complicate matters further, some volatiles can also impact the perception of sweetness and *vice versa* [8]. So far, we still do not really understand how all of these volatiles and non-volatiles are integrated into the unique flavor perception of a fruit. For breeding programs, screening for the large range of flavor chemicals is not practically possible. Therefore, it is important to characterize the molecular mechanisms and regulation of flavor in order to understand the complexity of this trait. Knowledge of biosynthetic pathways of fruit flavor compounds and regulatory mechanisms will lead to efficient breeding strategies, such as to identify markers that track flavor-associated chemicals.

Several studies in tomato, peach, strawberry and banana have been performed, identifying and characterizing the most important genes and encoded enzymes involved in aroma-related volatiles [9-14], however, very few studies have been carried out in citrus [15]. Although volatile constituents of flavor and aroma have been identified in tangerine [3,4,16], research on the mechanisms of regulation or modulation, especially in citrus, is very limited. Progress in gene isolation related to volatile production has been impeded by the lack of information concerning plant secondary metabolism, with flavor-associated volatiles [17]. Even for some of the most important metabolites, pathways for synthesis have only recently been established or remain to be established [18]. An integrated approach, including metabolomics, genomics, transcriptomics and proteomics, and determining fundamental metabolism, can make an important contribution toward this goal [2,19-22].

In the present study, we selected contrasting volatile and non-volatile profiles between two mandarin hybrids: Murcott and Temple. The two hybrids have similar genetic backgrounds due to having the same general parentage of mandarin and sweet orange, although their exact origins are unknown [23]. Despite that, both of these cultivars have good fruit flavor, although previous studies indicate that Temple is much richer in volatiles than Murcott, especially in sesquiterpenes and esters [4]. In addition to a comparison of volatile and non-volatile (sugars, acids, and carotenoids) compounds, and the interrelationships of these chemical components, a comparative iTRAQ (isobaric tags for relative and absolute quantification) proteome analysis was used to identify qualitative and quantitative differences in the proteome between the two hybrids at three levels of maturity. iTRAQ is a powerful approach, using isotope labeling coupled with multidimensional liquid chromatography and tandem mass spectrometry (MS), thereby enabling sensitive assessment and quantification of protein levels [24-26]. This analysis helped to better understand the pathways and genes controlling synthesis of flavor volatiles during mandarin hybrid fruit maturation, and to identify enzymes and genes involved in their biosynthesis pathways, especially concerning the terpenoid biosynthesis pathway.

## Results

### Differences in sugar, organic acid and carotenoid content between Murcott and Temple

**Figure 1:**
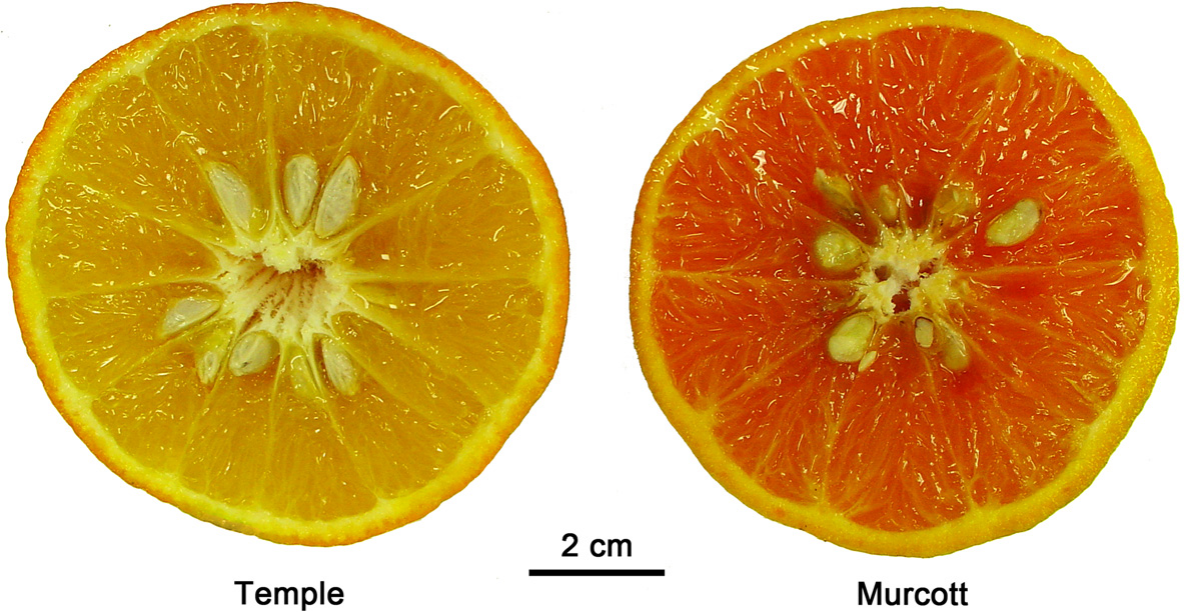
**Cross section of Temple and Murcott mandarin hybrid fruit.**

**Figure 2:**
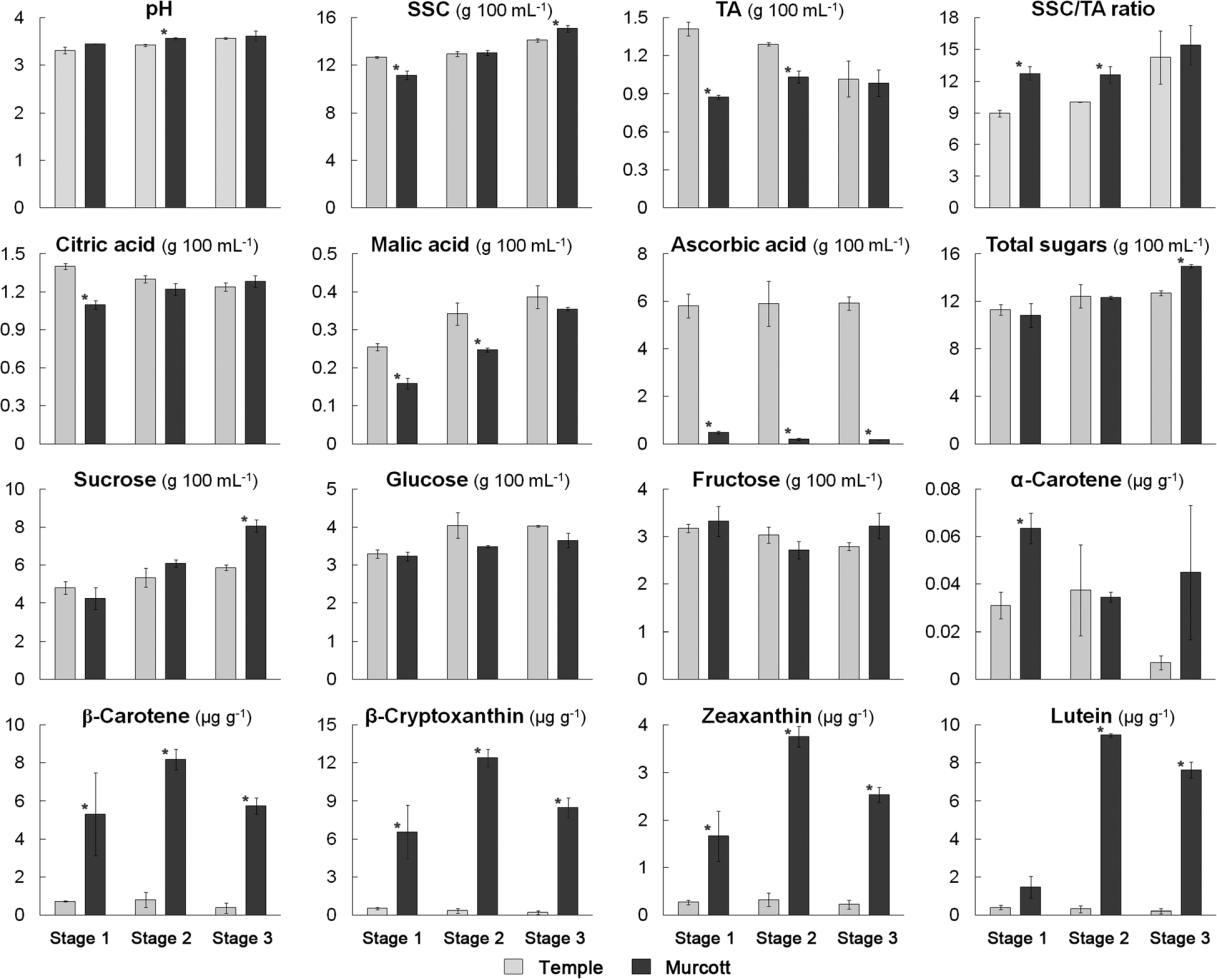
**Sugar, organic acid and carotenoid content in Temple and Murcott mandarin hybrid fruit at three developmental stages (stage 1: 22-Dec-2008; stage 2: 30-Jan-2009; and stage 3: 11-Mar-2009).** Student’s T-test was used to determine the statistical significance of the differences between mean values for Temple and Murcott at the same developmental stage; standard error bars are provided. *: significant difference (P < 0.05); SSC: soluble solids content; TA: titratable acidity.

Fruits of Temple and Murcott were different in flesh color (Figure 1). There were differences for sugars, organic acids and carotenoids between Temple and Murcott at the three maturity stages. Among sugars, only sucrose and total sugars were higher in Murcott than Temple at stage 3, and total soluble solids content (SSC) at stage 1 and 3. However, no differences were found in fructose and glucose. Among acids, Temple was higher than Murcott for citric acid at stage1, malic acid and titratable acidity (TA) at stage 1 and 2, and ascorbic acid at all three stages, respectively. The pH values for Temple were significantly lower at stage 2. Overall, ascorbic acid was 21 times higher in Temple than Murcott. SSC/titratable acidity (TA) was lower in Temple at stage 1 and 2. SSC/TA is an indicator of maturity in citrus, and no differences were found between the two cultivars in stage 3. All carotenoids, except α-carotene for stage 2 and 3 and lutein for stage 1, were significantly higher in Murcott than in Temple (Figure 2).

### Differences in aroma volatiles between Murcott and Temple

A total of 121 volatile compounds were detected by gas chromatography-mass spectrometry (GC-MS), with 108 compounds in Temple and 60 compounds in Murcott, respectively (Additional file 1: Table S1). Only 48 volatiles were found in both Temple and Murcott. There were 46 volatiles unique to Temple, in addition to 14 unknown compounds, whereas 12 volatiles were found only in Murcott (Table 1). The sum of total relative peak areas (peak area of compounds divided by peak area of internal standard) was twice as high in Temple than in Murcott, 21.9 for Temple, 11.5 for Murcott, respectively (Table 2). Terpenoid-related compounds contributed more than 85 and 95% of the total volatiles in Temple and Murcott respectively, also the volatile profile was markedly different. Valencene accounted for 9.4% of the total profile in Temple, whereas no valencene nor nootkatone was detected in Murcott. Sesquiterpenes were 0.15% and 3.10% and esters were 0.38% and 7.16% in Murcott and Temple, respectively. We found seven carotenoid-derived volatiles in Temple: nerol, neral, geranial, neryl acetate, α-ionone, geranyl acetone, and β-ionone. In contrast, only two of these, neryl acetate and geranyl acetone, were found in Murcott. D-limonene was the most abundant volatile compound which accounted for 80.8% and 64.4% of the volatile profile in Murcott and Temple, respectively. Murcott had two branched aldehydes, 3-methyl pentanal and 4-methyl hexanal, which were lacking in Temple. However, Temple had one branched alcohol, 3-methyl-1-butanol, and one branched ester, ethyl 2-methylbutyrate, likely to have been derived from the branched alcohol, whereas Murcott did not have these compounds (Table 2).

**Table 1:** **Volatiles in Temple and Murcott mandarin hybrid fruit arranged by chemical class**

**Temple only**	**Murcott only**	**Both**
**Monoterpenes**	**Monoterpenes**	**Monoterpenes**
Isoterpinolene	β-Pinene	α-Thujene
3-Carene	(+)-4-Carene	α-Pinene
2-Carene	**Aldehydes**	Sabinene
3-Methyl-4-methylenebicyclo[3.2.1]oct-2-ene	Butanal	β-Myrcene
	3-Methyl pentanal	α-Phellandrene
**Sesquiterpenes**	4-Methyl hexanal	γ-Terpinene
β-Elemene	ρ-Menth-1-en-9-al	ρ-Cymene
β-Cubebene	p-Menth-1-en-9-al isomer	d-Limonene
β-Humulene	**Ester**	β-Phellandrene
α-Caryophyllene	Ethyl acetate	γ-Terpiene
α-Selinene	**Ether**	ρ-Mentha-3,8-diene
γ-Selinene	Ethyl ether	Terpinolene
Valencene	**Hydrocarbons**	**Sesquiterpenes**
Aromadendrene	(E,E)-2,6-dimethyl-1,3,5,7-octatetraene	α-Cubebene
Calamenene		Copaene
(−)-α-Panasinsen	**Furans**	Caryophyllene
Eremophilene	2-n-Butyl furan	δ-Cadinene
Eudesma-3,7-diene	2-Pentyl furan	**Aldehydes**
4,11-Selinadiene		Acetaldehyde
**Aldehydes**		Propanal
(E)-2-Pentenal		Pentanal
Geranial (carotenoid)		Hexanal
Neral (carotenoid)		Heptanal
**Ketones**		Octanal
Acetone		Nonanal
Nootkatone		Decanal
α-Ionone (carotenoid)		(E)-2-Hexenal
β-Ionone (carotenoid)		(E)-2-Heptenal
**Alcohols**		(E)-2-Octenal
1-Hexanol		(E)-2-Nonenal
3-Methylbutanol		(E)-2-Decenal
(Z)-ρ-Mentha-2,8-dien-1-ol		Perillaldehyde
β-Terpineol		**Ketones**
Nerol (carotenoid)		1-Pentene-3-one
**Esters**		3-Pentanone
Ethyl butanoate		4-Heptanone
Ethyl 2-butenoate		d-Carvone
Ethyl 2-methylbutanoate		Dihydrocarvone
Ethyl pentanoate		Geranyl acetone (carotenoid)
Ethyl hexanoate		**Alcohols**
Ethyl-3-hydroxyhexanoate		Ethyl alcohol
Ethyl octanoate		1-Penten-3-ol
Propyl butanoate		Linalool
Methyl butanoate		Terpinen-4-ol
Methyl hexanoate		α-Terpineol
Hexyl acetate		**Esters**
Linalool acetate		Octyl acetate
Terpinyl acetate		Citronellol acetate
**Ether**		Neryl acetate (carotenoid)
1,8-Cineole		**Hydrocarbons**
**Hydrocarbons**		1,3-Pentadiene
(E)-2,6-Dimethyl-2,6-octadiene		(Z)-2,6-Dimethyl-2,6-octadiene
1,5-Dimethyl-cyclooctadiene		(+/−)-4-Acetyl-1-methylcyclohexene
**Furan**		
2-Ethyl furan		**Furan**
		2-Methyl furan

Carotenoid-derived volatiles are in parentheses.

**Table 2:** **Content of major volatile classes in Temple and Murcott mandarin hybrid fruit**

**Chemical class**	**Murcott**	**Temple**	**P value**
Aliphatic alcohols	0.045 ± 0.021	0.094 ± 0.043	0.356
Branched alcohols	n. d.	0.002 ± 0.001	
Aliphatic aldehydes	0.910 ± 0.257	0.755 ± 0.138	0.442
Branched aldehydes	0.005 ± 0.002	n. d.	
Aliphatic esters	0.044 ± 0.017	1.561 ± 0.246	0.000
Branched esters	n. d.	0.006 ± 0.001	
Aliphatic ketones	0.014 ± 0.001	0.019 ± 0.002	0.001
d-Limonene	9.266 ± 1.203	14.03 ± 2.317	0.110
Monoterpenes except d-Limonene	0.937 ± 0.141	1.323 ± 0.217	0.191
Valencene	n. d.	2.053 ± 0.367	
Sesquiterpenes except Valencene	0.017 ± 0.004	0.677 ± 0.004	0.000
Terpene alcohols	0.123 ± 0.013	0.720 ± 0.144	0.007
Terpene aldehydes	0.013 ± 0.003	0.026 ± 0.005	0.035
Terpene esters	0.011 ± 0.011	0.061 ± 0.010	0.004
Terpene ketones	0.057 ± 0.011	0.061 ± 0.010	0.764
Ethers	n. d.	0.348 ± 0.073	
Furans	0.022 ± 0.004	n. d.	
Other hydrocarbon	n. d.	0.149 ± 0.031	
Other	0.005 ± 0.001	0.007 ± 0.001	0.390
Total	11.47 ± 1.51	21.90 ± 3.000	0.030

Total ion current of target compound was divided by that of internal standard, 3-hexanone.

### Differentially expressed proteins in Temple versus Murcott

We identified 280 differentially expressed proteins in Temple versus Murcott (Additional file 1: Table S2). Of these identified proteins, 92 were significantly differentially expressed in juice sacs at the three ripening stages (fold change > 1.5, P < 0.05) (Table 3). We found 42, 54 and 45 expressed proteins in ripening stage 1, stage 2 and stage 3, respectively. There were 22 proteins in common between stage 1 and 2, 24 between stage 2 and 3, whereas only 9 proteins in common were identified between stage 1 and 3. Five proteins were present across all three stages: hypothetical protein (gi|225442225), superoxide dismutase (SOD) (gi|77417715), phospholipase D alpha (gi|169160465), plastid-lipid-associated protein (gi|62900641), and UDP-glucosyltransferase family 1 protein (gi|242199340). All proteins were more highly expressed in Murcott than Temple in stage 2, whereas most proteins were more highly expressed in Temple than Murcott in stage 1. In stage 3, 13 proteins were up-regulated versus 32 down-regulated in Temple versus Murcott. We found several important proteins involved in volatile production. Phospholipase D alpha (gi|169160465), a key enzyme involved in membrane deterioration which produces precursors to aliphatic alcohols and aldehydes, was up-regulated in Temple versus Murcott at stage 1, but not stage 2 and 3. The Family1 glycotranferases might affect biosynthesis and accumulation of glycosides that bind volatile terpenoids. Isopentenyl diphosphate Delta-isomerase I (gi|6225526) isomerizes isopentenyl diphosphate (IPP) to its isomer dimethylallyl diphosphate (DMAPP) and was up-regulated in Murcott versus Temple at ripening stage 2. Valencene synthase (gi|33316389) was the protein that was the most different between the two cultivars, being 25 times higher in Temple than in Murcott at ripening stage 3. Several proteins from the glycolysis pathway were identified: triosephosphate isomerase (gi|77540216), a triosphosphate isomerase-like protein (gi|76573375), and pyruvate decarboxylase (gi|17225598). All were only expressed in ripening stage 3, and were higher in Murcott than in Temple. A citrate synthase precursor (gi|624676) was found in ripening stage 1, upregulated in Temple in comparison with Murcott. In addition to citrus synthase, malate dehydrogenase (gi|27462762) and isocitrate dehydrogenase (gi|5764653) of the tricarboxylic acid (TCA) cycle were also found and downregulated in Temple versus Murcott. Glutamate decarboxylase (gi|70609690) and aspartate aminotransferase (gi|255551036), involved in glutamate synthesis, were also identified.

**Table 3:** **Differentially expressed proteins in fruit flesh of Temple (Te) versus Murcott (Mu) mandarin hybrid fruit**

**Accession**	**Name**	**Species**	**iTRAQ ratio fold change**					
			**Stage 1**		**Stage 2**		**Stage 3**	
			**Te/Mu**	**P value**	**Te/Mu**	**P value**	**Te/Mu**	**P value**
gi|11596186	cystatin-like protein	*Citrus x paradisi*	4.041	0.036	0.647	0.002		
gi|118061963	extracellular solute-binding protein, family 5	*Roseiflexus castenholzii* DSM 13941					0.483	0.047
gi|119367477	putative H-type thioredoxin	*Citrus cv.* Shiranuhi	10.782	0.001	0.404	0.001		
gi|119367479	putative cyclophilin	*Citrus cv.* Shiranuhi			0.588	0.037	2.347	0.002
gi|121485004	cytosolic phosphoglycerate kinase	*Helianthus annuus*	5.535	0.002				
gi|124360080	Galactose mutarotase-like	*Medicago truncatula*					1.724	0.003
gi|125546170	hypothetical protein OsI_14032	*Oryza sativa* Indica Group			0.561	0.014		
gi|14031067	dehydrin COR15	*Citrus x paradisi*					2.806	0.000
gi|147809484	hypothetical protein	*Vitis vinifera*			0.608	0.022	0.696	0.065
gi|147836508	hypothetical protein	*Vitis vinifera*					1.630	0.024
gi|147853192	hypothetical protein	*Vitis vinifera*					1.803	0.018
gi|15219028	26.5 kDa class I small heat shock protein-like	*Arabidopsis thaliana*					0.491	0.008
gi|15235730	phosphoenolpyruvate carboxykinase (ATP), putative/PEP carboxykinase, putative/PEPCK, putative	*Arabidopsis thaliana*					1.899	0.034
gi|159471948	U2 snRNP auxiliary factor, large subunit	*Chlamydomonas reinhardtii*			0.255	0.044		
gi|166850556	CTRSFT1-like protein	*Poncirus trifoliata*	3.261	0.011	0.237	0.005		
gi|169160465	phospholipase D alpha	*Citrus sinensis*	4.060	0.000	0.240	0.000	0.573	0.000
gi|17225598	pyruvate decarboxylase	*Fragaria x ananassa*					0.286	0.012
gi|183579873	chitinase	*Citrus unshiu*	1.534	0.012				
gi|192912988	40S ribosomal protein S4	*Elaeis guineensis*	1.601	0.049				
gi|218202932	14-3-3 protein	*Dimocarpus longan*			0.227	0.016		
gi|221327587	ascorbate peroxidase	*Citrus maxima*	4.863	0.000	0.180	0.049		
gi|2213425	hypothetical protein	*Citrus x paradisi*	0.627	0.000	0.524	0.001		
gi|223949137	unknown	*Zea mays*	5.116	0.003				
gi|224069008	predicted protein	*Populus trichocarpa*	6.992	0.001				
gi|224099429	predicted protein	*Populus trichocarpa*			0.587	0.014	0.316	0.002
gi|224109966	predicted protein	*Populus trichocarpa*			0.476	0.040		
gi|224127346	predicted protein	*Populus trichocarpa*			0.156	0.007	0.641	0.043
gi|224128794	predicted protein	*Populus trichocarpa*			0.298	0.007	0.382	0.022
gi|224135985	predicted protein	*Populus trichocarpa*			0.248	0.006	0.366	0.021
gi|225424861	PREDICTED: hypothetical protein isoform 2	*Vitis vinifera*					0.536	0.040
gi|225425914	PREDICTED: hypothetical protein	*Vitis vinifera*			0.429	0.002	0.425	0.010
gi|225439785	PREDICTED: hypothetical protein	*Vitis vinifera*			0.441	0.007	0.658	0.023
gi|225441981	PREDICTED: hypothetical protein	*Vitis vinifera*			0.304	0.002	0.568	0.007
gi|225442225	PREDICTED: hypothetical protein	*Vitis vinifera*	9.896	0.015	0.576	0.010	0.571	0.002
gi|225451968	PREDICTED: similar to mangrin	*Vitis vinifera*	4.507	0.040	0.263	0.095		
gi|231586	ATP synthase subunit beta	*Hevea brasiliensis*			0.134	0.004	0.555	0.007
gi|242199340	UDP-glucosyltransferase family 1 protein	*Citrus sinensis*	7.535	0.002	0.394	0.008	0.539	0.030
gi|255539613	phosphoglucomutase, putative	*Ricinus communis*			0.142	0.020		
gi|255543156	conserved hypothetical protein	*Ricinus communis*	7.967	0.000				
gi|255544686	eukaryotic translation elongation factor, putative	*Ricinus communis*			0.424	0.006	0.323	0.008
gi|255550111	heat-shock protein, putative	*Ricinus communis*	3.788	0.043				
gi|255551036	aspartate aminotransferase, putative	*Ricinus communis*					0.599	0.037
gi|255561582	Patellin-3, putative	*Ricinus communis*			0.588	0.017		
gi|255571742	peptidase, putative	*Ricinus communis*			0.275	0.004		
gi|255586766	monodehydroascorbate reductase, putative	*Ricinus communis*			0.429	0.003	0.493	0.001
gi|255641409	unknown	*Glycine max*			0.645	0.021		
gi|255642211	unknown	*Glycine max*	0.521	0.011	0.121	0.001		
gi|255644696	unknown	*Glycine max*	5.914	0.002				
gi|257659867	unnamed protein product	*Linum usitatissimum*			0.329	0.235	0.368	0.047
gi|257675725	unnamed protein product	*Zea mays*	3.832	0.019				
gi|257690969	unnamed protein product	*Citrus sinensis*			0.384	0.002		
gi|257712573	unnamed protein product	*Brassica napus*	9.086	0.011			0.664	0.006
gi|257720002	unnamed protein product	*Glycine max*			0.551	0.001	0.387	0.007
gi|257726687	unnamed protein product	*Zea mays*	1.650	0.035	0.387	0.001		
gi|27462762	malate dehydrogenase	*Lupinus albus*					0.305	0.003
gi|29124973	unknown	*Populus tremuloides*					2.039	0.031
gi|33316389	valencene synthase	*Citrus sinensis*					25.730	0.022
gi|33325127	eukaryotic translation initiation factor 5A isoform VI	*Hevea brasiliensis*					1.914	0.039
gi|33340236	copper/zinc superoxide dismutase	*Citrus limon*	3.706	0.001	0.638	0.004		
gi|37524017	COR15	*Citrus clementina x Citrus reticulata*	10.311	0.006			2.382	0.010
gi|3790102	pyrophosphate-dependent phosphofructokinase alpha subunit	*Citrus x paradisi*	1.724	0.025			0.554	0.011
gi|40646744	mitochondrial citrate synthase precursor	*Citrus junos*			0.201	0.032	0.553	0.018
gi|4580920	vacuole-associated annexin VCaB42	*Nicotiana tabacum*			0.209	0.046	0.330	0.007
gi|4704605	glycine-rich RNA-binding protein	*Picea glauca*	4.452	0.009				
gi|530207	heat shock protein	*Glycine max*	4.177	0.045				
gi|544437	Probable phospholipid hydroperoxide glutathione peroxidase	*Citrus sinensis*					3.140	0.039
gi|5764653	NADP-isocitrate dehydrogenase	*Citrus limon*			0.430	0.006	0.437	0.003
gi|6094476	Thiazole biosynthetic enzyme	*Citrus sinensis*			0.228	0.007		
gi|6166140	Elongation factor 1-delta 1	*Oryza sativa* Japonica Group	7.427	0.045	0.654	0.028		
gi|6225526	Isopentenyl-diphosphate Delta-isomerase I	*Clarkia breweri*			0.562	0.033		
gi|624674	heat shock protein	*Citrus maxima*						
gi|624676	citrate synthase precursor	*Citrus maxima*	2.731	0.020				
gi|62900641	Plastid-lipid-associated protein	*Citrus unshiu*	6.082	0.002	0.289	0.000	0.662	0.022
gi|63333659	beta-1,3-glucanase class III	*Citrus clementina x Citrus reticulata*			0.493	0.141	2.712	0.000
gi|6518112	H + −ATPase catalytic subunit	*Citrus unshiu*	4.754	0.017	0.598	0.007		
gi|6682841	sucrose synthase	*Citrus unshiu*	3.194	0.025	0.632	0.009		
gi|6682843	sucrose synthase	*Citrus unshiu*			0.144	0.008	0.575	0.024
gi|7024451	glycine-rich RNA-binding protein	*Citrus unshiu*	1.886	0.531				
gi|70609690	glutamate decarboxylase	*Citrus sinensis*	3.588	0.025	0.643	0.043		
gi|7269241	UDPglucose 4-epimerase-like protein	*Arabidopsis thaliana*	0.424	0.011	0.158	0.004		
gi|74486744	translation elongation factor 1A-9	*Gossypium hirsutum*	4.923	0.008				
gi|76573375	triosphosphate isomerase-like protein	*Solanum tuberosum*					0.311	0.000
gi|77417715	SOD	*Citrus maxima*	0.638	0.017	0.118	0.010	0.322	0.013
gi|77540216	triosephosphate isomerase	*Glycine max*					0.514	0.022
gi|77744899	temperature-induced lipocalin	*Citrus sinensis*	4.028	0.018	0.548	0.016		
gi|82623427	glyceraldehyde 3-phosphate dehydrogenase-like	*Solanum tuberosum*			0.661	0.297		
gi|862480	valosin-containing protein	*Glycine max*	1.510	0.029	0.374	0.010		
gi|870794	polyubiquitin	*Arabidopsis thaliana*	4.534	0.005				
gi|90820120	UDP-glucose pyrophosphorylase	*Cucumis melo*	7.835	0.028				
gi|9082317	actin	*Helianthus annuus*	3.959	0.051	0.527	0.001		
gi|9280626	UDP-glucose pyrophosphorylase	*Astragalus membranaceus*	9.821	0.002			1.626	0.022
gi|9757974	polyubiquitin	*Arabidopsis thaliana*			0.585	0.011		

The P value was selected from the most significant one among three biological replications. Additional file 1: Table S2 has the result from all three biological replications. Stage 1 was on December 22, 2008, Stage 2 was on January 30, 2009, and Stage 3 was on March 11, 2009.

Gene annotation was conducted using the Blast2GO program for all 92 identified proteins. The biological interpretation was further completed by assigning them to metabolic pathways using Kyoto Encyclopedia of Genes and Genomes (KEGG) annotation. KEGG analysis assigned the 46 differentially expressed proteins to 48 metabolic pathways (Additional file 1: Table S3). Most biosynthetic pathways identified were glycolysis, citric acid cycle, sugar synthesis, amino acid synthesis and terpene synthesis. Additional file 2: Figure S1 shows the distributions of GO terms (2^nd^ level GO terms) according to biological processes, cellular components and molecular function. Most differentially expressed proteins were predicted to be involved in carbohydrate, amino acid, and lipid metabolism as well as in energy production. We found 10 enzymes involved in the glycolysis pathway and 16 enzymes involved in different amino acid pathways (Table 4; Additional file 1: Table S3).

**Table 4:** **KEGG assigned differentially expressed proteins between Temple and Murcott mandarin hybrid fruit in metabolic pathways**

**KEGG pathway**	**Pathway**	**Enzyme number**
Carbohydrate metabolism	Amino sugar and nucleotide sugar metabolism	ec:2.7.7.9, ec:3.2.1.14, ec:5.1.3.2,ec:5.4.2.2
	Ascorbate and aldarate metabolism	ec:1.10.3.3, ec:1.11.1.11, ec:1.6.5.4
	Butanoate metabolism	ec:4.1.1.15
	Tricarboxylic acid cycle (TCA)	ec:1.1.1.37, ec:1.1.1.42, ec:2.3.1.12, ec:2.3.3.1, ec:4.1.1.49
	Fructose and mannose metabolism	ec:2.7.1.11, ec:2.7.1.90, ec:4.1.2.13,ec:5.3.1.1
	Galactose metabolism	ec:2.7.1.11, ec:2.7.7.9, ec:5.1.3.2, ec:5.4.2.2
	Glycerophospholipid metabolism	ec:3.1.4.4
	Glycolysis/Gluconeogenesis	ec:1.2.1.12, ec:2.3.1.12, ec:2.7.1.11, ec:2.7.2.3, ec:4.1.1.1, ec:4.1.1.49, ec:4.1.2.13, ec:5.1.3.3, ec:5.3.1.1, ec:5.4.2.2
	Glyoxylate and dicarboxylate metabolism	ec:1.1.1.37, ec:1.11.1.6, ec:2.3.3.1
	Pentose and glucuronate interconversions	ec:2.7.7.9, ec:3.1.1.11
	Pentose phosphate pathway	ec:1.1.1.49, ec:2.7.1.11, ec:4.1.2.13, ec:5.4.2.2
	Pyruvate metabolism	ec:1.1.1.37, ec:2.3.1.12, ec:4.1.1.49, ec:4.4.1.5
Amino acid metabolism	Alanine, aspartate and glutamate metabolism	ec:2.6.1.1, ec:2.6.1.2, ec:4.1.1.15
	Arginine and proline metabolism	ec:2.6.1.1, ec:3.5.3.1
	beta-Alanine metabolism	ec:4.1.1.15
	Cysteine and methionine metabolism	ec:2.6.1.1
	Glutathione metabolism	ec:1.1.1.42, ec:1.1.1.49, ec:1.11.1.11, ec:1.11.1.12, ec:1.11.1.15, ec:1.11.1.9, ec:2.5.1.18
	Phenylalanine metabolism	ec:1.11.1.7,ec:2.6.1.1
	Phenylalanine, tyrosine and tryptophan biosynthesis	ec:2.6.1.1
	Taurine and hypotaurine metabolism	ec:4.1.1.15
	Tryptophan metabolism	ec:1.11.1.6
	Tyrosine metabolism	ec:2.6.1.1
	Valine, leucine and isoleucine degradation	ec:2.3.1.168
Other secondary metabolites	Isoquinoline alkaloid biosynthesis	ec:2.6.1.1
	Novobiocin biosynthesis	ec:2.6.1.1
	Tropane, piperidine and pyridine alkaloid biosynthesis	ec:1.11.1.6
	Streptomycin biosynthesis	ec:5.4.2.2
Energy metabolism	Carbon fixation in photosynthetic organisms	ec:1.1.1.37, ec:2.6.1.1, ec:2.6.1.2, ec:2.7.2.3, ec:4.1.1.49, ec:4.1.2.13, ec:5.3.1.1
	Carbon fixation pathways in prokaryotes	ec:1.1.1.37, ec:1.1.1.42
	Inositol phosphate metabolism	ec:5.3.1.1
	Methane metabolism	ec:1.1.1.37, ec:1.11.1.6, ec:1.11.1.7, ec:2.7.1.11, ec:4.1.2.13
	Oxidative phosphorylation	ec:3.6.3.6
Lipid metabolism	alpha-Linolenic acid metabolism	ec:5.3.99.6
	Arachidonic acid metabolism	ec:1.11.1.9
	Ether lipid metabolism	ec:3.1.4.4
	Primary bile acid biosynthesis	ec:1.3.1.3
	Steroid degradation	ec:1.1.1.145
	Steroid hormone biosynthesis	ec:1.1.1.145, ec:1.3.1.3
Metabolism of terpenoids and polyketides	Terpenoid backbone biosynthesis	ec:5.3.3.2
Nucleotide metabolism	Arginine and proline metabolism	ec:3.5.3.11
	Cysteine and methionine metabolism	ec:4.4.1.14
	Purine metabolism	ec:3.6.1.3, ec:5.4.2.2
Xenobiotics biodegradation and metabolism	Chlorocyclohexane and chlorobenzene degradation	ec:3.1.1.45
	Drug metabolism - cytochrome P450	ec:2.5.1.18
	Fluorobenzoate degradation	ec:3.1.1.45
	Metabolism of xenobiotics by cytochrome P450	ec:2.5.1.18
	Toluene degradation	ec:3.1.1.45

## Discussion

In this study, two thirds of differentially expressed proteins were identified in the pathways of glycolysis and TCA as well as amino acid, sugar and starch metabolism (Tables 3 and 4). This is understandable, because the upstream precursors for most volatiles come from carbohydrate metabolism, mainly through sugar and starch metabolism through the glycolysis pathway, which is important for providing the carbon skeleton and toward the different branches that lead to the aforementioned volatiles. Most organic acids, amino acids, terpenes and fatty acids are produced from glycolysis and TCA. For amino acids, the carbon skeletons are derived from 3-phosphoglycerate, phosphoenolpyruvate or pyruvate generated in glycolysis, or from 2-oxoglutarate and oxaloacetate generated in TCA [20]. Terpenoids are enzymatically synthesized de novo from acetyl CoA and pyruvate provided by the carbohydrate pools in plastids and the cytoplasm [27].

The differences in protein expression between Temple and Murcott were due to the different ripening patterns of these two hybrids. Temple is a middle-late variety whereas Murcott is a very late variety; however in Florida citrus production conditions, and depending on season, Temple and Murcott maturity times may overlap. These differences in time of maturity might explain proteins being more highly expressed in Temple than Murcott in stage 1, whereas all proteins were more highly expressed in Murcott than Temple in stage 2, and mixed protein expression levels were seen in stage 3. Feng *et al.* [28] found that glutamate decarboxylase (gi|70609690) was one of two proteins likely associated with carbohydrate and acid metabolism in the ripening fruit. In our study, this protein is expressed more in Temple at stage 1, but less in stage 2 than Murcott. This might also explain the differences in levels of volatiles, sugar, organic acids in different stages between Temple and Murcott.

### Sugar, TCA and glycolysis biosynthesis

Sucrose is the major sugar translocated in the plant, the major photo-assimilate stored in the plant, and can be degraded by cell wall sucrose synthase to glucose and fructose. Glucose can be converted into pyruvate, generating small amounts of adenosine triphosphate (ATP) and nicotinamide adenine dinucleotide reduced form (NADH) via the glycolysis pathway. Glucose phosphomutase (gi|255539613, EC 5.4.2.2) was down-regulated in Temple in stage 2, and is an enzyme responsible for the conversion of D-glucose 1-phosphate into D-glucose 6-phosphate. Sucrose synthase (gi|6682841/gi|6682843, EC 2.4.1.13) catalyzes the degradation of sucrose into UDP-glucose and fructose, up-regulated in Temple at stage 1 and down-regulated in stage 2 and 3. The high expression of sucrose synthase in Murcott stage 2 might partially explain why Murcott had higher sucrose than Temple (Figure 2). Sucrose, in turn, is derived from hexose phosphates through UDP-glucose pyrophosphorylase, (gi|90820120, gi|9280626, EC 2.7.7.9). The glycolysis biosynthesis is a central pathway that produces important precursor metabolites: six-carbon compounds of glucose-6P and fructose-6P and three-carbon compounds of glycerone-P, glyceraldehyde-3P, glycerate-3P, phosphoenolpyruvate, and pyruvate. Acetyl-CoA and another important precursor metabolite are produced by oxidative decarboxylation of pyruvate. The reaction, mediated by phosphofructokinase (gi|3790102, EC 2.7.1.11), is one of the key control points of glycolysis in plants. This reaction catalyzes the interconversion of fructose-6-phosphate and fructose-1, 6-bisphosphate.

Citric acid is the main organic acid in citrus fruit juice. Yun *et al.* [29] found citric acid comprised up to 90% of the total organic acid content throughout the entire postharvest period. Citrate may be utilized by three major metabolic pathways for sugar production, amino acid synthesis, and acetyl-CoA metabolism. 2-Oxoglutarate can be then metabolized to an amino acid such as glutamate. Six enzymes acting in the TCA cycle were identified in our study including: pyruvate decarboxylase (gi|17225598, EC 4.1.1.1), malate dehydrogenase (gi|27462762, EC 1.1.1.37), isocitrate dehydrogenase (NADP+) (gi|5764653, EC 1.1.1.42), dihydrolipoyllysine-residue acetyltransferase (gi|225442225, EC 2.3.1.12), citrate synthase (gi|624676, EC 2.3.3.1) and phosphoenolpyruvate (PEP) carboxykinase (gi|15235730, EC 4.1.1.49). The pyruvate decarboxylase enzyme, down-regulated in Temple, links the TCA cycle to glycolysis. Plant cells can convert PEP to malate via oxaloacetate in reactions catalyzed by PEP carboxykinase (gi|15235730, EC 4.1.1.49) and malate dehydrogenase (gi|27462762, EC 1.1.1.37) [1]. Citrate can be produced by condensation of oxaloacetate and acetyl-CoA, catalyzed by citrate synthase which was up-regulated in Temple in stage 2. Citrate synthase is the rate-limiting enzyme of the TCA cycle [29]. The result might explain the higher citric acid content in Temple than Murcott. The oxidative decarboxylation of isocitrate into 2-oxoglutarate is mediated by the action of isocitrate dehydrogenase. The last step of the TCA pathway is the interconversion of malate to oxaloacetate utilizing nicotinamide adenine dinucleotide oxidized form (NAD+) /NADH and is catalyzed by malate dehydrogenase. In general, however, the changes of enzymes in the TCA cycle and glycolysis cannot fully explain the difference of organic acid and sugar contents in Temple compared to Murcott. Katz *et al*. [21] indicated that changes in metabolite amounts in fruit do not always correlate well with protein expression levels, reflecting the complication of regulated pathway outputs.

### Amino acids, oxidization, ascorbate-glutathione cycle

KEGG pathway analysis conducted by Blast2GO indicated that seven enzymes are involved in the glutathione metabolic pathway (Table 4). In plants, glutathione is crucial for biotic and abiotic stress management. It is a pivotal component of the glutathione-ascorbate cycle, a system that reduces poisonous hydrogen peroxide. Pan *et al.* [30] found that expression levels of five antioxidative enzymes (catalase, peroxidase, ascorbate peroxidase, glutathione reductase and superoxide dismutase) were altered in a mutant orange “Hong Anliu” which has a high level of lycopene, and implied a regulatory role of oxidative stress on carotenogenesis. In our study, the protein expression of L-ascorbate peroxidase (gi|221327587, EC 1.11.1.11), phospholipid-hydroperoxide glutathione peroxidase (gi|544437, EC 1.11.1.12), superoxide dismutase (SOD) (gi|77417715), and monodehydroascorbate reductase (gi|255586766, EC 1.6.5.4), were mixed (Table 3). SOD and monodehydroascorbate reductase had lower expression in Temple, whereas, other proteins were higher in stage 1 and 3, and lower in stage 2 (Table 3). We could not define a clear relationship between antioxidative enzyme activity and the amount of carotenoids. The discrepancy is likely due to other regulatory pathways, since there are many steps involved in the biosynthesis pathways that are tightly regulated [31]. Liu *et al.* [32] found glutamate decarboxylase is an enzyme catalyzing the conversion of L-glutamate to γ-aminobutyric acid, and suggested that it is possible that glutamate decarboxylase (gi|70609690) could participate in regulating the cytosolic pH.

### Volatile biosynthesis

**Figure 3:**
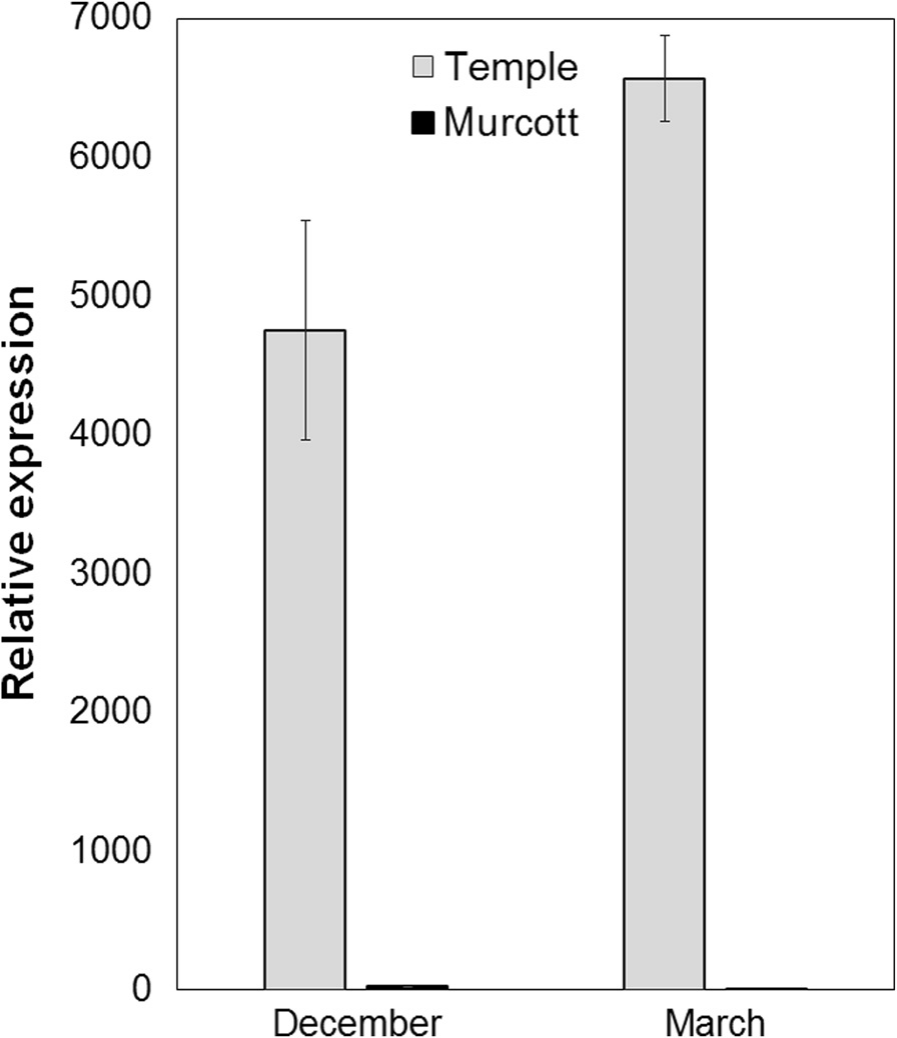
**QRT-PCR validation of the expression profiles of** ***Cstsp1*** **genes at two time points.** Results were expressed relative to the value of the expression of Murcott *Cstps1* in March.

All terpenoids derive from the common building units isopentenyl diphosphate (IPP) and its isomer dimethylallyl diphosphate (DMADP). Both IPP and DMADP are synthesized via two parallel pathways, the mevalonate (MVA) pathway, which is active in the cytosol, and the methylerythritol 4-phosphate (MEP) pathway, which is active in the plastids. In this study, IPP isomerase (gi|6225526) upregulated in Murcott relative to Temple, catalyzes isomerization between IPP and dimethylallyl diphosphate (Table 3). Aharoni *et al.* [33] found that the pool of IPP in the plastids might affect the formation of sesquiterpenes in the cytosol given that transport of isoprenoid precursors is known to occur from the plastids to the cytosol. A valencene synthase (gi|33316389) expression explains the difference in valencene content between Temple and Murcott. Sharon-Asa *et al.* [15] isolated and characterized the valencene synthase gene (*Cstps1*) and reported that valencene accumulates during the ripening of Valencia orange fruits together with *Cstps1*. Results from the current work agreed with their study (Additional file 2: Figure S2-A). In order to validate the result, real-time PCR showed that the gene expression of *Cstps1* was found to be over 217 and 2720 times higher in Temple than in Murcott on Dec 22, 2008 and March 11, 2009, respectively (Figure 3). Murcott expression of *Cstps1* gene is very severely reduced.

Non-volatile sugar conjugates constitute a large pool of precursors for many of the important flavor volatiles. Enzymes synthesizing and hydrolyzing these sugar conjugates are likely to influence the volatile profiles. Family 1 glycosyltransferases (gi|242199340), often referred to as UDP glycosyltransferases, is the largest in the plant kingdom [34], which catalyze the transfer of a glycosyl moiety from UDP-sugars to a wide range of acceptor molecules. Glycosyltransferase might affect biosynthesis and accumulation of glycosides of volatile terpenoids. Fan *et al.* [35] identified three putative terpenoid UDP-glycosyltransferase (UGT) genes in sweet orange. The different expression of glycotranferase family 1 in three stages of fruit ripening in Temple might explain the difference in terpenoid volatile levels compared with Murcott.

Fatty acids play a major role in ester volatile synthesis. We have identified the phospholipid D (gi|169160465, EC 3.1.4.4) in all three ripening stages. Oke *et al.* [36] found that the transgenic tomato fruits with an antisense phospholipase D (PLD) showed improved red color, lycopene content, and results suggest that a reduction in PLD activity may lead to increased membrane stability and preservation of membrane compartmentalization that can have positive quality impacts for transgenic fruit and their products. We did not find major enzyme differences downstream, such as the lipoxygenase (LOX) pathway, which comprises the action of phospholipase, lipoxygenase, and hydroxyperoxide. The lipid-derived volatiles represent the bulk of aroma volatiles in tomato and are generated by the lipoxygenase (LOX) pathway [37]. In addition, pyruvate decarboxylase (gi|17225598) is believed to be involved in the pathway that provide aldehydes and alcohols for ester synthesis [38].

### Correlation between valencene/sesquiterpenes accumulation and total carotenoids

**Figure 4:**
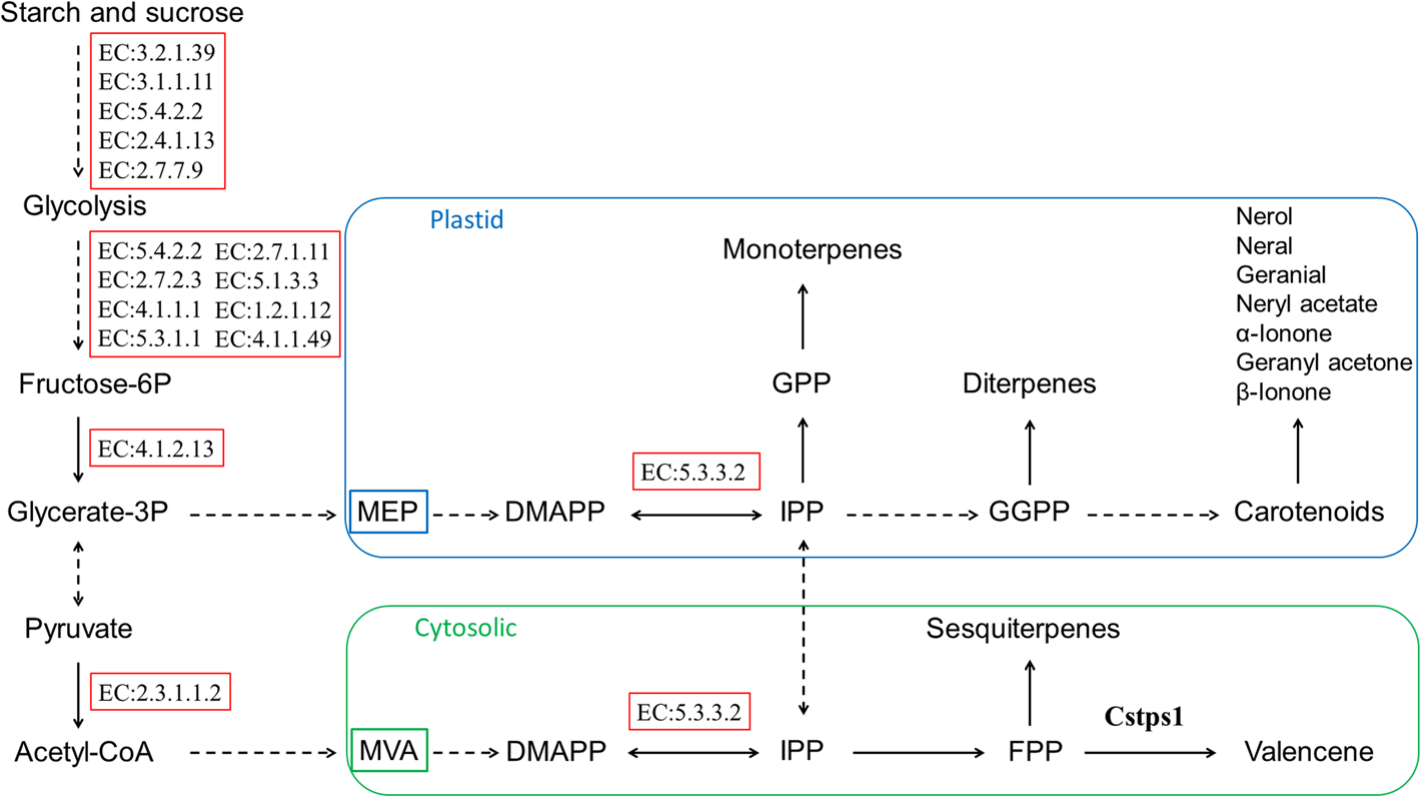
**Summary of metabolic pathways leading to terpenoid-associated volatile synthesis.** The differently expressed KEGG enzymes between Temple and Murcott mandarin hybrid fruit are in red boxes. The second metabolites are presented in yellow boxes. Pathway names are presented in the blue box. In most cases, arrows indicate multiple enzyme reactions. Abbreviations: MEP, 2-C-methyl-D-erythritol 4-phosphate; MVA, mevalonate; IPP, isopentenyl diphosphate; DMAPP, dimethyl-allyl diphosphate; GPP, geranyl diphosphate; FPP, farnesyl diphosphate; GGPP, geranylgeranyl diphosphate; Cstps1,valencene synthase.

It is generally recognized that the cytosolic MVA pathway is responsible for the synthesis of sesquiterpenes, phytosterols and ubiquinone, whereas monoterpenes, gibberellins, abscisic acid, carotenoids and the prenyl moiety of chlorophylls, plastoquinone and tocopherol are produced in plastids via the MEP pathway [27,39]. Although the subcellular compartmentation of MVA and MEP pathways allows them to operate independently, metabolic “crosstalk” between the two pathways was prevalent, particularly in the direction of plastids to cytosol [5] (Figure 4). Prenyltransferase condenses dimethylallyl diphosphate with two IPP molecules to produce FPP or three IPP to geranylgeranyl diphosphate (GGPP). In this study, Temple, had lower carotenoids but higher number of apocarotenoid volatiles than Murcott (Additional file 1: Table S1). Davidovich-Rikanati *et al.* [11] indicated that a transgenic tomato expressing a monoterpene synthesis gene resulted in lighter color in comparison with wild type tomatoes. Because GGPP is the precursor of the carotenoids, the activity of valencene synthase (*Cstps1*) converting FPP to valencene could be one of the limiting steps for carotenoid production in Temple (Figure 4). The important flavor volatile genes are those that encode enzymes responsible for synthesis of the end products and those encoding factors that regulate pathway output [18]. Valencene synthase (*Cstps1*) is the protein for synthesis of the end product, valencene. Klee *et al.* [18] indicated that all of the apocarotenoid volatile QTLs identified to date are associated with carotenoid biosynthetic enzymes, and substrate availability rather than enzyme synthesis appears to be limiting apocarotenoid volatiles. Our study indicated that the high concentration of carotenoids in Murcott might be due to its lack of valencene synthase activity (Figure 3; Additional file 2: Figure S2-B) as well as less sesquiterpenes and other carotenoid derived volatiles (Additional file 1: Table S1), compared with Temple. In tomato and watermelon, studies have indicated that carotenoid pigmentation patterns have profound effects on apocarotenoid volatile compositions [40,41]. By comparison with Murcott, our results suggest that the diversion of high valencene and other sesquiterpenes into the terpenoid pathway together with high production of apocarotenoid volatiles might have resulted in the lower concentration of carotenoids in Temple.

## Conclusions

Two thirds of differently expressed proteins were identified in the pathway of glycolysis and TCA, as well as amino acid, sugar and starch metabolism. This highlights the importance of these metabolic pathways for providing the carbon skeleton of the upstream precursors for most volatiles. Total carotenoids were significantly higher and apocarotenoid volatiles lower in Murcott than in Temple. It appears that high concentrations of apocarotenoid volatile compounds may result in low concentrations of carotenoids in Temple. In addition, we found that valencene synthase (*Cstps1*) was severely reduced in Murcott, and consequently, no valencene was detected in Murcott fruit during development, while substantial amounts were present in Temple. Further study is needed to confirm if there is a relationship between carotenoid concentrations and apocarotenoid volatile compounds, sesquiterpenes such as valencene, in citrus fruit. Improving fruit flavor is a challenging task using classic breeding methods because of the difficulty in scoring and quantifying such a complex trait. An increased understanding of biosynthetic pathways for fruit flavor compounds and corresponding regulatory mechanisms will lead to more efficient breeding strategies to improve flavor.

## Methods

### Plant material

Fruit of Murcott and Temple cultivars were collected on three harvest dates (designated as Stage 1, 2, and 3 respectively): 22 December 2008, 30 January 2009, and 11 March 2009 from groves at the University of Florida, Citrus Research and Education Center (UF-CREC) (Figure 1). These trees were grown under the same environmental conditions of soil, irrigation and illumination. Fruit maturity for Murcott and Temple was determined based on previous results [4], and three years of measurements of volatiles and non-volatiles at different stages amoung 14 mandarin hybrids including Temple and Murcott. Sample fruits were also selected based on fruit of similar size, color, and flavor by experienced breeders. Both Temple and Murcott have the same rootstock, Cleopatra mandarin, and are grown in the center part of field. In total, 20 fruits were collected randomly around the tree, 10 fruits for protein and 10 fruits for volatile compound identification, respectively. Three to four fruits were bulked as biological replications for proteome analysis.

### Sugars, organic acids and carotenoids analysis

The measurement of sugars and acids was based on the method described by Baldwin *et al.* [42]. For titratable acidity (TA) and soluble solids content (SSC), TA was determined by titrating to pH8.2 with 0.1 M NaOH using an autotitrator (Mettler Toledo DL50, Columbus, OH) and SSC using a refractometer (Atago PR-101, Tokyo, Japan). Individual sugar and acid analysis was performed via high performance liquid chromatography (HPLC). Approximately 40 g of juice was extracted using 70 mL of an 80% ethanol/deionized water solution. The mixture was boiled for 15 min, cooled, and filtered (Whatman #4 filter paper, Batavia, IL). The filtered solution was brought to 100 mL with 80% ethanol. A total of 10 mL of the filtered solution was then passed through a C^18^ Sep-Pak (Waters/Millipore), followed by a 0.45 μm Millipore (Siemens-Millipore, Shrewbury, MA) filter. Individual sugars analysis was performed by HPLC with a refractive index detector (Perkin Elmer, Norwalk, Conn) equipped with a Waters Sugar Pak column [43-45]; The mobile phase was 10^−4^ M ethylenediaminetetraacetic acid disodium calcium salt (CaEDTA) (0.5 mL min^−1^ flow rate at 90°C). All results are expressed as g 100 mL^−1^ juice. Organic acids, including ascorbic acid, were analyzed using a Perkin-Elmer Series 200 auto sampler (Waltham, MA), a Spectra System P4000 pump, and a Spectra System UV 6000 LP detector (Thermo Fisher Scientific, Waltham, MA). Acids were separated on an AltechOA1000 Prevail organic acid column with a flow rate of 0.2 mL min^−1^ at 35°C and a mobile phase of 0.01 N H_2_SO_4_ [42,46]. The injection volume was 20 μL.

Carotenoids in the pellet and supernatant were analyzed using HPLC. Juice samples (30 mL) were centrifuged at 10,000 × g for 15 min. The pellet extracts were collected by dissolving pellets in acetone. Both pellet extracts and supernatants were individually filtered through a 0.45 μm filter into amber vials and stored at −20°C until injected into an HPLC (20 μL loop) equipped with an YMC carotenoid column (YMC Co. Ltd., Komatsu City, Japan). Elution conditions included a three-solvent gradient composed initially of water/methanol/methyl tertbutyl ether (4/81/15, v/v/v), and changed to linear gradients of 4/6/90 (v/v/v) by 60 min at a flow rate of 1 mL min^−1^, at 30°C. Compounds were detected using a photo diode array (PDA) detector scanning 200–700 nm at 5 nm increments, identified using standards (Sigma, Carotenoid Nature) and quantified using absorbance measurements. Values for pellet extracts and supernatants were then added together for each sample.

### Volatile compound identification

Sample preparation for volatile and aroma identification used the same methods as previously described [4]. Briefly, Temple and Murcott samples were juice composites of 10 fruits with 2 replications of 5 fruits. The fruit were washed, rinsed and gently juiced manually using a table-top manual juicer (model 3183; Oster, Rye, NY, USA) to avoid potential peel components (peel oil) entering the juice. Juice samples (2.5 mL) were placed in 20 mL glass vials (Gerstel, Inc., Baltimore, MD, USA) along with saturated sodium chloride solution (2.5 mL) to help drive volatiles into the headspace and inhibit any potential enzymatic activity. An internal standard (3-hexanone, 1 ppm) was added to juice samples. The vials were capped and stored at −20°C until analyzed. The extraction of aroma volatiles was performed using solid-phase microextraction (SPME) with an MPS-2 auto sampler (Gerstel). The vials were incubated at 40°C for 30 min and volatile compounds were identified by comparison of their mass spectra with library entries (NIST/EPA/NIH Mass Spectral Library, version 2.0; National Institute of Standards and Technology, Gaithersburg, MA, USA), as well as by comparing retention indices (RIs) with published RIs on both columns. Volatiles were semi-quantified by dividing peak area with the peak area of the internal standard.

### Statistical analysis of volatile and non-volatile compounds

Two pooled samples from ten fruits were used for each harvesting time. All calculations were based on means of harvesting time. The differences of volatile and non-volatile compounds between Temple and Murcott were examined by an analysis of variance using the PROC GLM procedure of the SAS 9.4 statistical software package (http://www.sas.com).

### Protein extraction

Protein extraction was modified based on the following description [21]. Briefly, the juice sacs were ground in homogenization buffer containing 0.5 M MOPS-KOH pH 8.5, 1.5% PVPP, 7.5 mM EDTA, 2 mM DTT, 0.1 mM PMSF, and 0.1% (v/v) protease inhibitor cocktail (Sigma, St. Louis, MO, USA). The homogenates were filtered through four layers of cheesecloth and centrifuged at 1500 × g for 20 min to eliminate cellular debris and nuclei. The pellet was discarded and the supernatant was centrifuged at 12000 × g for 20 min at 4°C. Soluble protein was precipitated in ammonium sulfate (85%) and collected by centrifugation at 12000 × *g*. The pellets were resuspended in a buffer containing 10 mM KH_2_PO_4_ and 0.5 mM DTT and desalted with a PD-10 column (Amersham Bioscience, GE Healthcare, Piscataway, NJ, USA) according to manufacturer’s instruction. Protein concentration was determined using the Bio-Rad Bradford protein assay (Bio-Rad, Hercules, CA. USA). One hundred μg protein from each sample was precipitated in 80% cold acetone at −20°C overnight, centrifuged at 18,000 rpm for 20 min at 4°C, and washed once with 80% cold acetone.

### iTRAQ Labeling and data analysis

In total, 18 samples were labeled and analyzed (2 cultivars × 3 maturity levels × 3 replications). Three to four fruits were pooled with 100 μg protein as one replication. iTRAQ labeling and data analysis were performed as a service by the Interdisciplinary Center for Biotechnology Research (ICBR) Proteomic Core facility at the University of Florida (Gainesville, FL, USA). For protein digestion, iTRAQ labeling and cation exchange were done according to the company’s protocols and described by Zhu *et al.* [47]. Briefly, the MS/MS data were analyzed by a thorough search considering biological modifications against the NCBI subset of green plants fasta database (downloaded on November, 2010) using the Paragon™ Algorithm of PROTEINPILOT v3.0 software suite (Applied Biosystems). For relative quantification of proteins, only MS/MS spectra unique to a particular protein and for which the sum of the signal-to-noise ratio for all of the peak pairs was greater than 9 were used for quantification (Applied Biosystems). To be identified as being differentially expressed, a protein had to be quantified with at least three spectra, a p < 0.05, and a ratio -fold change of at least 2 in more than two independent experiments (i.e. at least six peptides). Protein identities were confirmed using BLAST at the NCBI. Gene ontology analysis of identified proteins was carried out using Blast2GO [48]. The biological interpretation of the differentially expressed proteins was further completed by assigning them to metabolic pathways using Kyoto Encyclopedia of Genes and Genomes (KEGG) annotation. For proteins identified more than once, only the most significant identified protein was selected. In addition, functional classification of total identified proteins was analyzed by Blast2Go with default parameters (https://blast2go.com).

### RNA extraction and quantitative real-time reverse transcription polymerase chain reaction (QRT-PCR)

Total RNA from each sample was extracted using Trizol (Ambion), and contaminating DNA was eliminated using the Turbo DNA-free Kit (Ambion, Austin, TX). The concentration of RNA was measured in a NanoDrop ND-1000 spectrophotometer (NanoDrop Technologies, Wilmington, DE). Total RNA was diluted as 5 ng/μL^−1^. QRT-PCR was carried out in the Agilent Mx3005P System (Agilent Technology) using a Brilliant III Ultra-Fast SYBR Green QRT-PCR Master Mix (Agilent Technology). Glyceraldehyde 3-phosphate dehydrogenase (GAPDH) was used as a reference gene to provide relative quantification for the target gene valencene synthase (*Cstps1*). Primer sequences of *Cstps1* were used according to Sharon-Asa *et al.* [15] (Additional file 2: Table S4). The results represent normalized mean values and standard error of mean analyzed by using the program in the Agilent Mx3005P System.

### Availability of supporting data

The data supporting the results of this article are included within the article.

## Additional files

Additional file 1:
**The excel spread sheet contains 3 tables describing identified volatiles, proteins and results of metabolite pathway analyses in Temple and Murcott.** **Table S1.** Volatiles identified in Temple and Murcott mandarin hybrid fruit. **Table S2.** Total proteins identified in fruit flesh of Temple and Murcott mandarin hybrid fruits, and ratio of Temple versus Murcott. **Table S3.** Metabolite pathways containing differentially expressed proteins between Temple and Murcott mandarin hybrid fruits. Additional file 2:
**Detailed information on primers used for amplifying valencene synthase, gene ontology assignment, valencene and carotenoid content during fruit ripening in Temple and Murcott.** **Table S4.** Primers used for amplifying valencene synthase and control genes for real-time PCR. **Figure S1.** Gene Ontology (GO) assignment (2nd level GO terms) of differential proteins between Murcott and Temple. The differential proteins were categorized based on GO annotation and the proportion of each category was displayed according to: Biological process (A), Cellular component (B) and Molecular function (C). Because a gene could be assigned to more than one GO term, the sum of genes in a category would be above the total number 92. X axis indicates number of different expressed proteins. **Figure S2.** (A) Valencene production during fruit ripening in Temple and Murcott; (B) Carotenoid content in Temple and Murcott during ripening.

## Abbreviation

ATP: Adenosine triphosphate
Cstps1: Valencene synthase
CaEDTA: Ethylenediaminetetraacetic acid disodium calcium salt
DMPP: Isomer dimethylallyl diphosphate
GC-MS: Gas chromatography–mass spectrometry
GO: Gene ontology
HPLC: High performance liquid chromatography
IPP: Isopentenyl diphosphate
iTRAQ: Isobaric tags for relative and absolute quantification
KEGG: Kyoto encyclopedia of genes and genomes
MEP: Methylerythritol 4-phosphate
MVA: Mevalonate
NAD+: Nicotinamide adenine dinucleotide (oxidized form)
NAPDH: Nicotinamide adenine dinucleotide (reduced form)
QRT-PCR: Quantitative real-time Reverse transcription polymerase chain reaction
RIs: Retention indices
SOD: Superoxide dismutase
SPME: Solid-phase microextraction
SSC: Soluble solids content
TA: Titratable acidity
TCA: The tricarboxylic acid cycle

## References

[1] Baldwin IT (2010). Plant volatiles. Curr Biol.

[2] Tietel Z, Plotto A, Fallik E, Lewinsohn E, Porat R (2011). Taste and aroma of fresh and stored mandarins. J Sci Food Agric.

[3] Miyazaki T, Plotto A, Baldwin EA, Reyes-De-Corcuera JI, Gmitter FG (2012). Aroma characterization of tangerine hybrids by gas-chromatography-olfactometry and sensory evaluation. J Sci Food Agric.

[4] Miyazaki T, Plotto A, Goodner K, Gmitter FG (2011). Distribution of aroma volatile compounds in tangerine hybrids and proposed inheritance. J Sci Food Agric.

[5] Dudareva N, Klempien A, Muhlemann JK, Kaplan I (2013). Biosynthesis, function and metabolic engineering of plant volatile organic compounds. New Phytol.

[6] Goff SA, Klee HJ (2006). Plant volatile compounds: sensory cues for health and nutritional value?. Science.

[7] Tieman D, Bliss P, McIntyre LM, Blandon-Ubeda A, Bies D, Odabasi AZ (2012). The chemical interactions underlying tomato flavor preferences. Curr Biol.

[8] Baldwin EA, Goodner K, Plotto A (2008). Interaction of volatiles, sugars, and acids on perception of tomato aroma and flavor descriptors. J Food Sci.

[9] Beekwilder J, Alvarez-Huerta M, Neef E, Verstappen FW, Bouwmeester HJ, Aharoni A (2004). Functional characterization of enzymes forming volatile esters from strawberry and banana. Plant Physiol.

[10] Aharoni A, Keizer LCP, Bouwmeester HJ, Sun ZK, Alvarez-Huerta M, Verhoeven HA (2000). Identification of the SAAT gene involved in strawberry flavor biogenesis by use of DNA microarrays. Plant Cell.

[11] Davidovich-Rikanati R, Sitrit Y, Tadmor Y, Iijima Y, Bilenko N, Bar E (2007). Enrichment of tomato flavor by diversion of the early plastidial terpenoid pathway. Nat Biotechnol.

[12] Gonzalez Aguero M, Troncoso S, Gudenschwager O, Campos Vargas R, Moya Leon MA, Defilippi BG (2009). Differential expression levels of aroma-related genes during ripening of apricot (*Prunus armeniaca* L.). Plant Physiol Bioch.

[13] Zhang B, Shen JY, Wei WW, Xi WP, Xu CJ, Ferguson I (2010). Expression of genes associated with aroma formation derived from the fatty acid pathway during peach fruit ripening. J Agric Food Chem.

[14] Lucker J, Bowen P, Bohlmann J (2004). Vitis vinifera terpenoid cyclases: functional identification of two sesquiterpene synthase cDNAs encoding (+)-valencene synthase and (−)-germacrene D synthase and expression of mono- and sesquiterpene synthases in grapevine flowers and berries. Phytochemistry.

[15] Sharon-Asa L, Shalit M, Frydman A, Bar E, Holland D, Or E (2003). Citrus fruit flavor and aroma biosynthesis: isolation, functional characterization, and developmental regulation of Cstps1, a key gene in the production of the sesquiterpene aroma compound valencene. Plant J.

[16] Tietel Z, Feldmesser E, Lewinsohn E, Fallik E, Porat R (2011). Changes in the transcriptome of 'Mor' mandarin flesh during storage: emphasis on molecular regulation of fruit flavor deterioration. J Agric Food Chem.

[17] Muhlemann JK, Klempien A, Dudareva N (2014). Floral volatiles: from biosynthesis to function. Plant Cell Environ.

[18] Klee HJ (2010). Improving the flavor of fresh fruits: genomics, biochemistry, and biotechnology. New Phytol.

[19] Pan Z, Zeng Y, An J, Ye J, Xu Q, Deng X (2012). An integrative analysis of transcriptome and proteome provides new insights into carotenoid biosynthesis and regulation in sweet orange fruits. J Proteomics.

[20] Katz E, Fon M, Eigenheer RA, Phinney BS, Fass JN, Lin D (2010). A label-free differential quantitative mass spectrometry method for the characterization and identification of protein changes during citrus fruit development. Proteome Sci.

[21] Katz E, Fon M, Lee YJ, Phinney BS, Sadka A, Blumwald E (2007). The citrus fruit proteome: insights into citrus fruit metabolism. Planta.

[22] Guillaumie S, Fouquet R, Kappel C, Camps C, Terrier N, Moncomble D et al. Transcriptional analysis of late ripening stages of grapevine berry. BMC Plant Biol. 2011;11:165. 10.1186/1471-2229-11-165 PMC3233516 22098939

[23] Saunt J (2000). Citrus Varieties of the World: an Illustrated Guide.

[24] Chen S, Harmon AC (2006). Advances in plant proteomics. Proteomics.

[25] Gan CS, Chong PK, Pham TK, Wright PC (2007). Technical, experimental, and biological variations in isobaric tags for relative and absolute quantitation (iTRAQ). J Proteome Res.

[26] Pierce A, Unwin RD, Evans CA, Griffiths S, Carney L, Zhang L (2008). Eight-channel iTRAQ enables comparison of the activity of six leukemogenic tyrosine kinases. Mol Cell Proteomics.

[27] Schwab W, Davidovich-Rikanati R, Lewinsohn E (2008). Biosynthesis of plant-derived flavor compounds. Plant J.

[28] Feng C, Chen M, Xu CJ, Bai L, Yin XR, Li X (2012). Transcriptomic analysis of Chinese bayberry (*Myrica rubra*) fruit development and ripening using RNA-Seq. BMC Genomics.

[29] Yun Z, Li WY, Pan ZY, Xu J, Cheng YJ, Deng XX (2010). Comparative proteomics analysis of differentially accumulated proteins in juice sacs of ponkan (*Citrus reticulata*) fruit during postharvest cold storage. Postharvest Biol Tec.

[30] Pan ZY, Liu Q, Yun Z, Guan R, Zeng WF, Xu Q (2009). Comparative proteomics of a lycopene-accumulating mutant reveals the important role of oxidative stress on carotenogenesis in sweet orange (*Citrus sinensis* [L.] osbeck). Proteomics.

[31] Trindade H (2010). Molecular biology of aromatic plants and spices. A Rev Flavour Frag J.

[32] Liu Q, Zhu A, Chai L, Zhou W, Yu K, Ding J (2009). Transcriptome analysis of a spontaneous mutant in sweet orange [*Citrus sinensis* (L.) Osbeck] during fruit development. J Exp Bot.

[33] Aharoni A, Jongsma MA, Bouwmeester HJ (2005). Volatile science? Metabolic engineering of terpenoids in plants. Trends Plant Sci.

[34] Yonekura-Sakakibara K, Hanada K (2011). An evolutionary view of functional diversity in family 1 glycosyltransferases. Plant J.

[35] Fan J, Chen C, Yu Q, Li ZG, Gmitter FG (2010). Characterization of three terpenoid glycosyltransferase genes in 'Valencia' sweet orange (*Citrus sinensis* L. Osbeck). Genome.

[36] Oke M, Pinhero RG, Paliyath G (2003). The effects of genetic transformation of tomato with antisense phospholipase D cDNA on the quality characteristics of fruits and their processed products. Food Biotechnol.

[37] Pirrello J, Regad F, Latche A, Pech JC, Bouzayen M (2009). Regulation of tomato fruit ripening. CAB Reviews.

[38] Song J, Forney CF (2008). Flavour volatile production and regulation in fruit. Canadian J Plant Sci.

[39] Dudareva N, Negre F, Nagegowda DA, Orlova I (2006). Plant volatiles: recent advances and future perspectives. Crit Rev Plant Sci.

[40] Lewinsohn E, Sitrit Y, Bar E, Azulay Y, Ibdah M, Meir A (2005). Not just colors – carotenoid degradation as a link between pigmentation and aroma in tomato and watermelon fruit. Trends Food Sci Tech.

[41] Lewinsohn E, Sitrit Y, Bar E, Azulay Y, Meir A, Zamir D (2005). Carotenoid pigmentation affects the volatile composition of tomato and watermelon fruits, as revealed by comparative genetic analyses. J Agric Food Chem.

[42] Baldwin E, Plotto A, Manthey J, McCollum G, Bai J, Irey M (2010). Effect of Liberibacter infection (huanglongbing disease) of citrus on orange fruit physiology and fruit/fruit juice quality: chemical and physical analyses. J Agric Food Chem.

[43] Baldwin EA, Nisperos-Carriedo MO, Baker R, Scott JW (1991). Quantitative analysis of flavor parameters in six Florida tomato cultivars (*Lycopersicon esculentum* Mill). J Agric Food Chem.

[44] Baldwin EA, Scott JW, Einstein MA, Malundo TMM, Carr BT, Shewfelt RL (1998). Relationship between sensory and instrumental analysis for tomato flavor. J Am Soc Hortic Sci.

[45] Baldwin EA, Goodner K, Plotto A, Pritchett K, Einstein M (2004). Effect of volatiles and their concentration on perception of tomato descriptors. J Food Sci.

[46] Baldwin EA, Bai J, Plotto A, Cameron R, Luzio G, Narciso J (2012). Effect of extraction method on quality of orange juice: hand-squeezed, commercial-fresh squeezed and processed. J Sci Food Agric.

[47] Zhu M, Dai S, McClung S, Yan X, Chen S (2009). Functional differentiation of Brassica napus guard cells and mesophyll cells revealed by comparative proteomics. Mol Cell Proteomics.

[48] Conesa A, Gotz S, Garcia-Gomez JM, Terol J, Talon M, Robles M (2005). Blast2GO: a universal tool for annotation, visualization and analysis in functional genomics research. Bioinformatics.

