# Supplementary material for: Proteomic and metabolomic analyses provide insight into production of volatile and non-volatile flavor components in mandarin hybrid fruit
Source: BMC Plant Biol. 2015 Mar 6;15:76. doi: 10.1186/s12870-015-0466-9 (PMC4356138; doi:10.1186/s12870-015-0466-9)
Supplement: Additional file 2: — Detailed information on primers used for amplifying valencene synthase, gene ontology assignment, valencene and carotenoid content during fruit ripening in Temple and Murcott. Table S4. Primers used for amplifying valencene synthase and control genes for real-time PCR. Figure S1. Gene Ontology (GO) assignment (2nd level GO terms) of differential proteins between Murcott and Temple. The differential proteins were categorized based on GO annotation and the proportion of each category was displayed according to: Biological process (A), Cellular component (B) and Molecular function (C). Because a gene could be assigned to more than one GO term, the sum of genes in a category would be above the total number 92. X axis indicates number of different expressed proteins. Figure S2. (A) Valencene production during fruit ripening in Temple and Murcott; (B) Carotenoid content in Temple and Murcott during ripening. [file 12870_2015_466_MOESM2_ESM.docx]

**Table S4** Primers used for amplifying valencene synthase and control genes for real-time PCR.

| Gene Detected | Forward Primer (5’ 3’) | Reverse Primer (5’ 3’) |
| --- | --- | --- |
| GAPDH | GGAAGGTCAAGATCGGAATCAA | CGTCCCTCTGCAAGATGACTCT |
| CSTPS1 | CCCAGGCGTTGTACTTCATCA | CGACACGAGGCACTGAAAGA |

**Figure S1** Gene Ontology (GO) assignment (2nd level GO terms) of differential proteins between Murcott and Temple. The differential proteins were categorized based on GO annotation and the proportion of each category was displayed according to: Biological process (A), Cellular component (B) and Molecular function (C). Because a gene could be assigned to more than one GO term, the sum of genes in a category would be above the total number 92. X axis indicates number of different expressed proteins.

**Figure S2** (A) Valencene production during ripening in Temple and Murcott; (B) Carotenoid content in Temple and Murcott during ripening.
